# One Dimensional(1D)-to-2D Crossover of Spin Correlations in the 3D Magnet ZnMn_2_O_4_

**DOI:** 10.1038/srep17771

**Published:** 2015-12-08

**Authors:** S. M. Disseler, Y. Chen, S. Yeo, G. Gasparovic, P. M. B. Piccoli, A. J. Schultz, Y. Qiu, Q. Huang, S.-W. Cheong, W. Ratcliff

**Affiliations:** 1 NIST Center for Neutron Research, National Institute of Standards and Technology, Gaithersburg, Maryland 20899; 2 Department of Materials Science and Engineering, University of Maryland, College Park, Maryland 20742; 3 Rutgers Center for Emergent Materials and Department of Physics and Astronomy, Rutgers University, Piscataway, New Jersey 08854; 4 Intense Pulsed Neutron Source, Argonne National Laboratory, Argonne, Illinois 60439; 5 Korea Atomic Energy Research Institute, Daejeon, Korea

## Abstract

We report on the intriguing evolution of the dynamical spin correlations of the frustrated spinel ZnMn_2_O_4_. Inelastic neutron scattering and magnetization studies reveal that the dynamical correlations at high temperatures are 1D. At lower temperature, these dynamical correlations become 2D. Surprisingly, the dynamical correlations condense into a quasi 2D Ising-like ordered state, making this a rare observation of two dimensional order on the spinel lattice. Remarkably, 3D ordering is not observed down to temperatures as low as 300 mK. This unprecedented dimensional crossover stems from frustrated exchange couplings due to the huge Jahn-Teller distortions around Mn^3+^ ions on the spinel lattice.

Geometrically frustrated magnets, in which the magnetic interactions are frustrated by the geometry of the lattice, have been the subject of intense experimental and theoretical study^1^,^2^. Geometrical frustration has resulted in a number of intriguing ground states ranging from spin ice^3^ to spin liquids^4^,^5^. In the spinel structure, magnetic ions form corner-shared tetrahedra resulting in a spin liquid state in ZnCr_2_O_4_, for example^4^,^5^, while others order and may become multiferroic^6^. Through the use of ionic ordering, it has been possible to create quasi one dimensional(D) chains in the spinel lattice in systems such as LiCuVO_4_ 7, however, this is achieved only through severe modification of the lattice. In contrast, in the ZnV_2_O_4_ system orbital ordering gives rise to a system of tangled 1D chains^8^. In this work, we demonstrate that geometrical frustration stabilizes low dimensional magnetism in the spinel, ZnMn_2_O_4_. In this particular example, the system develops short range 1D dynamical correlations at room temperature. As the temperature is lowered, these correlations extend to two dimensions and finally the system achieves 2D magnetic order at 60 K. 2D ordering is extremely rare on the spinel lattice and only has been proposed in powder studies of isostructural Li_2_Mn_2_O_4_ 9 where the ordering pattern reported was completely different. Long range 3D order is not established even by 300 mK in ZnMn_2_O_4_.

Later, we will argue that the out of plane interactions in this lattice remain frustrated, resulting in two dimensional ordering in the lattice. The interactions within a given layer consists of weakly coupled spin chains with a relatively strong intrachain coupling resulting from direct exchange. This can be mapped onto the rectangular Ising model, considered by Onsager^10^. Onsager and others^10^,^11^,^12^ found that when the interchain coupling is on the order of 100 times weaker than that of the intrachain coupling, the essential physics is that of 1D chains. However, the presence of weak interchain coupling will allow the system to order magnetically. Dimensional crossovers from 2D-3D and 1D-3D have been studied in the past. However there is a paucity of studies of 1D-2D crossovers^11^. Here, we present an experimental realization of a system evincing this crossover.

At high temperatures, ZnMn_2_O_4_ crystallizes as a cubic spinel. However, this state is unstable and at 1323 K, a Jahn-Teller phase transition takes the system from the Fd
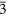
m cubic phase into a tetragonal I4_1_/amd phase with 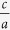 ratio of 1.62^13^,^14^. The Jahn Teller effect could have lowered the electronic energy of the system through the lone 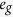 electron either occupying the 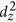 orbital, or the 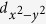 orbital, which would result in either elongated or contracted oxygen octahedra. Theory and experiment reveal that it is the 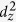 which is lower in energy^13^,^14^. Furthermore, theory suggests that these 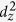 orbitals are ferro-ordered^15^. This orbital ordering will then favor 1D correlations along the chains through direct exchange. Thus, the orbital ordrering partially lifts the frustration of this lattice and allows us a rare opportunity to examine the ground state of uniaxial spins placed on a spinel lattice with anisotropic exchange interactions.

## Results

### Magnetization and Specific Heat

**Figure 1:**
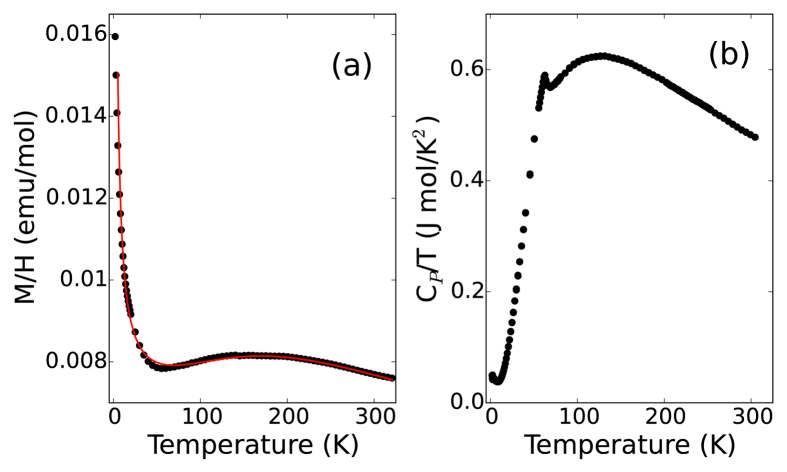
(**a**) Specific heat measurements on a powder of ZnMn_2_O_4_ indicating a transition at ≈60 K. (**b**) Susceptibility measurements showing low dimensional physics, (1 emu = 10^−3^ A/m). Solid (red) line is fit to classical model for a 1-D spin chain as described by Eq. 1.

We start our exploration of this system with measurements of the specific heat and magnetization of polycrystalline samples, shown in Fig. 1. The magnetization shows a broad maxima at high temperature, characteristic of systems with low dimensional ordering or correlations^11^,^12^. This behavior is well described by the functional form of one one-dimensional chains^16^, with the addition of a small Curie-term at low-temperatures. The magnetic susceptibility 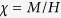 above 5 K is fit to this phenomenological function in the form of Eq. 1 using a Bayesian fitting routine^17^, where *J* is the average exchange interaction along the chains and 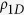 is the effective volume fraction of the sample exhibiting 1D-like susceptibility.


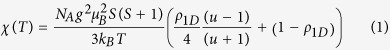


where, 
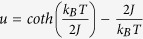
.

The result of this fit is shown as the solid (red) line in Fig. 1a with 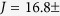 0.3 meV and 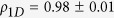. Here 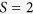 was fixed to represent the high-spin configuration of Mn^3+^. The small effective volume of the Curie-term indicates that the origin is either a minor impurity phase or result of a powder-averaged single-ion anisotropy that is not included this 1D model. Thus, despite the 3D nature of the crystalline lattice, we find that the primary magnetic correlations are low dimensional over a large range of temperatures.

The specific heat shown in Fig. 1b, also exhibits a broad maxima above 100 K, in addition to a small peak at *T* ≈ 60 K, corresponding to the minimum of the magnetic susceptibility. The peak is indicative of a phase transition in spatial dimensions greater than one^12^,^18^, suggesting a more complex ground state than simple chains. Due to the high temperature of the transition, it is generally ill advised to attempt to use models to obtain the magnetic contribution to the specific heat. Also, due to the Jahn-Teller transition, it is not possible to use an isostructural analogue to subtract the phonon contribution to the specific heat. Thus, this data can only be used to show consistency with the transition temperature determined by neutron scattering.

### Neutron Diffraction

**Figure 2:**
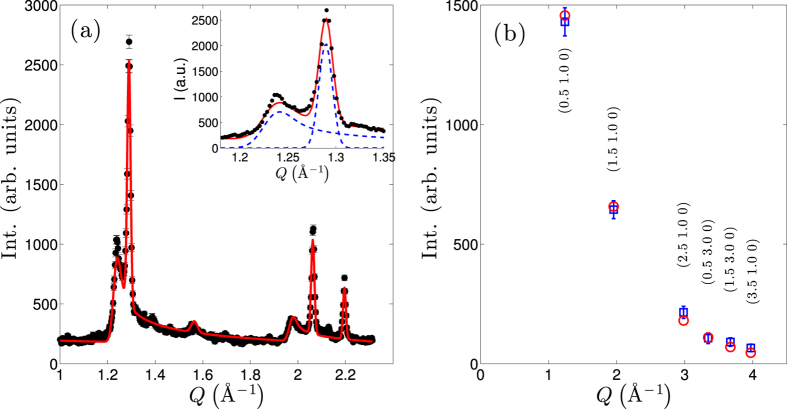
(**a**) Refinement of the powder at 300 mK taken on the BT1 spectrometer, the solid (red) line is the fit as described in the text. (inset) Close up of the low angle magnetic and nuclear peak. Dashed (blue) lines are fits of the individual asymmetric magnetic and gaussian nuclear peak, the solid line is the complete fit including background. (**b**) Refinement of the magnetic structure from single crystal neutron diffraction. Squares represent measurements, and circles represent calculated values for the model shown in Fig. 3. Error bars in this paper reflect counting statistics.

We turn now to neutron diffraction to directly study the onset of magnetic order and subsequent correlations. Powder diffraction measurements shown in Fig. 2a show asymmetric Warren lineshapes for magnetic peaks^19^,^20^ with a propagation vector 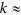 (0.5, 1, 0), indicative of 2D ordering with weak correlations along the c-axis. This is observed below the ordering temperature of 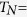 64 K, down to 300 mK. The asymmetric lineshapes of the magnetic peak at 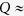 1.24 Å^−1^ is in stark contrast to the sharp, symmetric nuclear peak at 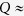 1.29 Å^−1^ highlighted in the inset of Fig. 2a. Reitveld refinement of high-resolution neutron diffraction patterns above 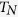 do not show any evidence for structural disorder or impurity phases, nor is there evidence for site-mixing of Zn and Mn ions beyond the 2% statistical error of the refinement. Thus, the disorder-free system clearly demonstrates that the magnetic structure remains 2D-like even though the crystalline structure is fully three-dimensional.

**Figure 3:**
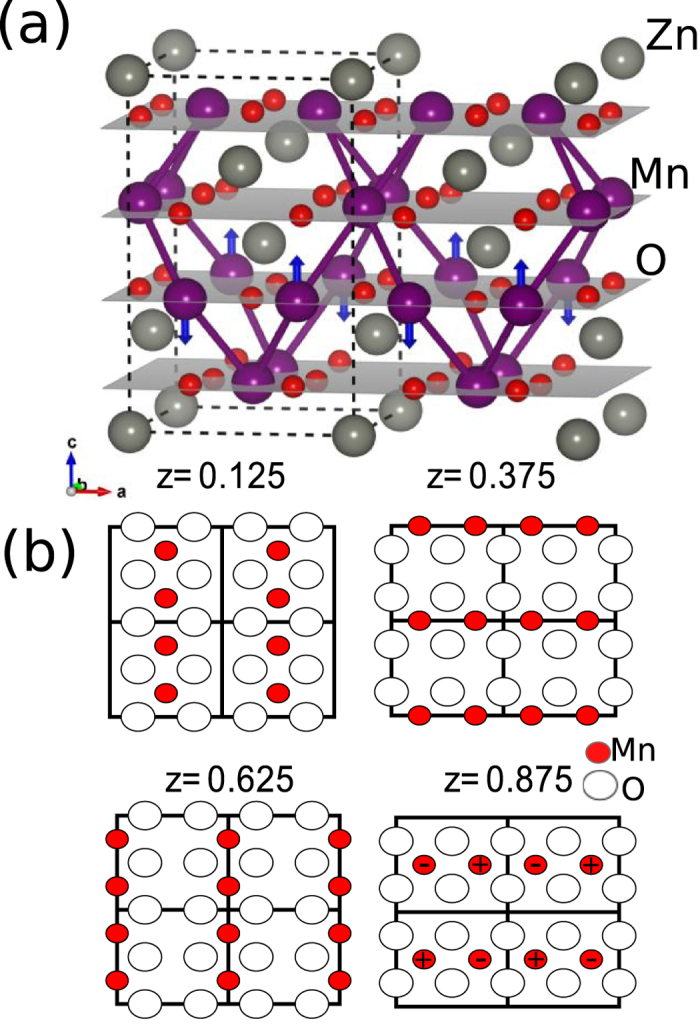
(**a**) Cartoon of the crystallographic and magnetic structure. Bonds highlighting interplane frustration bewtwen Mn-O layers. (**b**) Schematic of the magnetic structure of ZnMn_2_O_4_ in the **a-b** plane, with emphasis on the Mn and O atoms. (+) signs indicate spins out of the **a-b** plane and (−) signs represent spins into plane.

The effective correlation length within the 2D layers is determined by fitting the 300 mK data with a series of Warren lineshape magnetic peaks with a uniform correlation length. Gaussian peaks representing the nuclear peaks were also fit simultaneously. The magnetic structure factors were calculated from the magnetic structure shown in Fig. 3, that was later verified using single crystal diffraction. From this fit, shown as the solid line in Fig. 2a, we find that the in-plane magnetic correlation length is 200 ± 20 Å. A similar value for the correlation length was found at 40 K indicating that the correlation length is largely temperature independent below 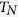.

Single crystal measurements performed on SCD^21^ show rods of scattering parallel to the c-axis, with ordering wave-vector k = (0.526, 1, 0) demonstrating 2D order similar to the powder material. We first solve this magnetic structure in long wavelength approximation k ≈ (0.5, 1, 0), noting that the difference corresponds to oscillations of the spins with very long periodicity approaching the correlation lengths derived from powder diffraction measurements. This propagation vector and rod-like scattering reveals that the individual layers shown in Fig. 3b order independently of each other. As the strongest coupling is through the direct exchange, we assume that the coupling is antiferromagnetic along the chains in each layer. Under this assumption we fit 6 magnetic reflections shown in Fig. 2b with the spin, S, as the only refineable parameter. We have restricted ourselves to a single quadrant to avoid possible systematic errors. We find *S* = 2.10 ± 0.04, which is consistent with that expected for the fully orbitally quenched moment. The 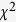 for this fit is 1.4 demonstrating excellent agreement between the data and this long-wavelength model. Fitting the data instead with the fully incommensurate model does not improve the quality of fit, and *S* is unchanged within error. Therefore, as the two models cannot be distinguished from one another, we will discuss the remainder of the results in the long-wavelength approximation of a commensurate magnetic structure.

In Fig. 3a, we show the spinel lattice along with a schematic of the magnetic structure in this commensurate approximation. In this image one can easily distinguish the magnetically frustrated bonds between layers leading to the quasi-2D structure observed low temperature. The magnetic lattice can be viewed as consisting of chains of spins, alternating between the (1 0 0) and (0 1 0) directions as one moves along the c-axis, as demonstrated in Fig. 3b.

**Figure 4:**
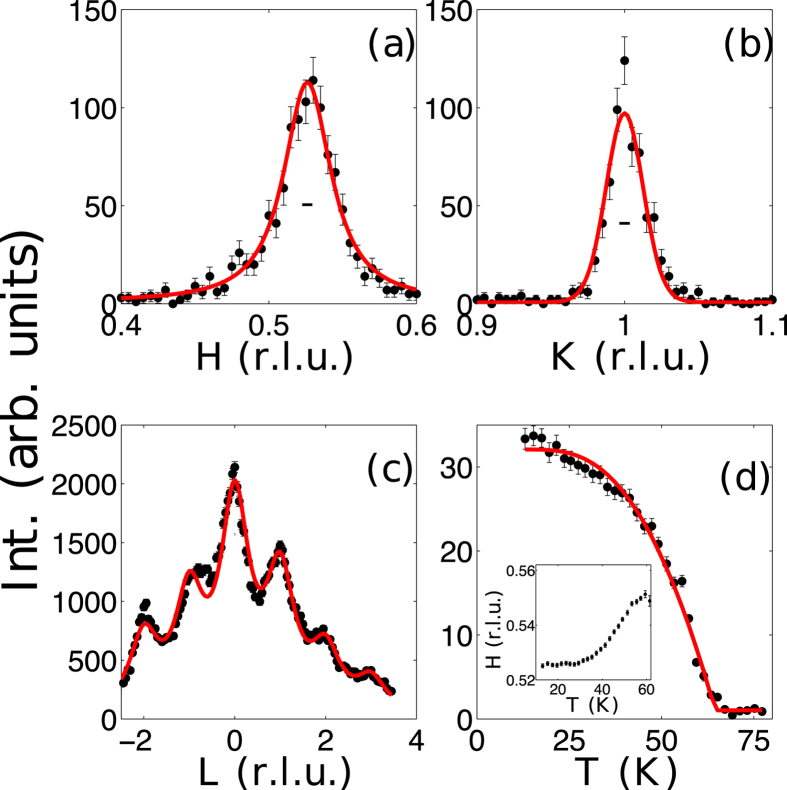
(**a**) Correlations measured between chains measured at 10 K. (**b**) Correlations measured along chains measured at 10 K. (**c**) Correlations measured along a rod of scattering at (0.526 1 L) at 4.5 K. Solid (red) lines are fits as described in the text. (**d**) Temperature dependent integrated intensity of the (∼0.526, 1, 0) magnetic reflection. It is fit to a mean field order parameter. (Inset) The center position of the (H 1 0) reflection as a function of temperature.

The temperature dependent integrated intensity of the (0.526, 1, 0) magnetic reflection is shown in Fig. 4d. A fit using a mean field order parameter shown as the solid line gives an ordering temperature 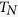 = 64.5 ± 0.2 K in agreement with the peak observed in specific heat. The small intensity above the transition stems from critical scattering resulting from correlated fluctuations in the paramagnetic state. The commensurability of the magnetic structure determined by the location of the magnetic peak along *H*, shown in the inset of Fig. 4d, is observed to monotonically increase with decreasing temperatures below 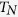. This change in commensurability can be described as an increase in the periodicity for the small spin amplitude modulations between chains, and likely results from competing interactions beyond the nearest neighbor. Although this presents an interesting perturbation to the ordered state, this incommensurability is not necessary in understanding the fundamental dynamics and emergent low dimensionality, as we will show.

In Fig. 4, we examine the correlation lengths of the single crystal sample in the magnetically ordered phase. Measurements along the *H* and *K* directions were performed at 10 K, while measurements along *L* were measured at 4.5 K. For the rod at (0.5 1 L), scans along *H* correspond to interchain correlations and scans along *K* correspond to intrachain correlations. Fig. 4a,b show fits to a 2D lorentzian convolved with the instrumental resolution denoted by the horizontal bar in the respective figure. These fits give an interchain correlation length of 47.6 ± 3.4  and an intrachain correlation length of 398.2 ± 163.4 Å. Fig. 4c shows the weak correlations along the c-axis. For alignment purposes we measured the rod in the (*H* 2*H L*) zone, however, the rod is actually at (0.526 1 0), thus as we scan along *L*, the fall will be steeper than the magnetic form factor because our scan will increasingly deviate from the true direction of the rod. Thus, instead of fitting to a sum of lattice lorentzians, we simply sum a series of lorenzians centered at integer *L* positions. This results in an interplane correlation length of 3.88 ± 0.04 Å, or approximately the distance between Mn-containing planes. This demonstrates that correlations are effectively 2D within the 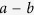 plane in agreement with that expected from our model of the magnetic structure.

### Inelastic Neutron Scattering

**Figure 5:**
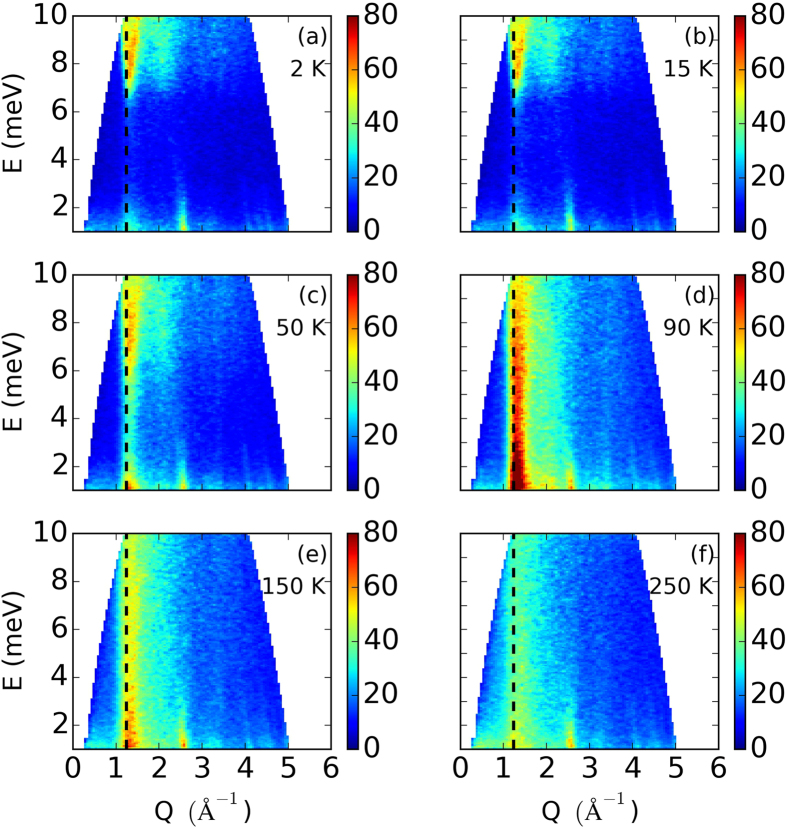
Overview of excitations in the system at various temperatures, measured in powders on DCS. The dashed line at 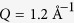 represents the location of the first magnetic peak also investigated in Fig. 6.

Inelastic neutron scattering directly probes of the spin-dynamics and excitations in the magnetically ordered state, as well as the low-dimensional spin-spin correlations above 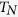. In Fig. 5, we show DCS measurements performed on powders to provide an overview of excitations in this system. Well below 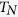 (Fig. 5a,b) the spin-excitations are completely gapped, with an energy gap Δ ∼ 6 meV at 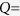 1.2 Å^−1^, corresponding to the (0.526,1,0) magnetic reflection. This spin-gap is indicative of the single-ion anisotropy expected in the Jahn-Teller distorted structure. The well-defined magnon band extends beyond the observable energy range here, demonstrating large exchange interactions exist between spins as proposed by our fits of the magnetic susceptibility. At higher temperatures the gap becomes less well defined (Fig. 5c) before becoming a continuum of scattering in the paramagnetic region above 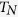. This narrow band of scattering remains centered above 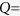 1.2 Å^−1^ magnetic peak, and weaken with increasing temperature (4d to 4f). Even at 250 K–several times 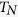
–significant scattering remains at high energies above this magnetic wavevector, making evident strong correlations between spins for finite times, i.e. short-range order.

**Figure 6:**
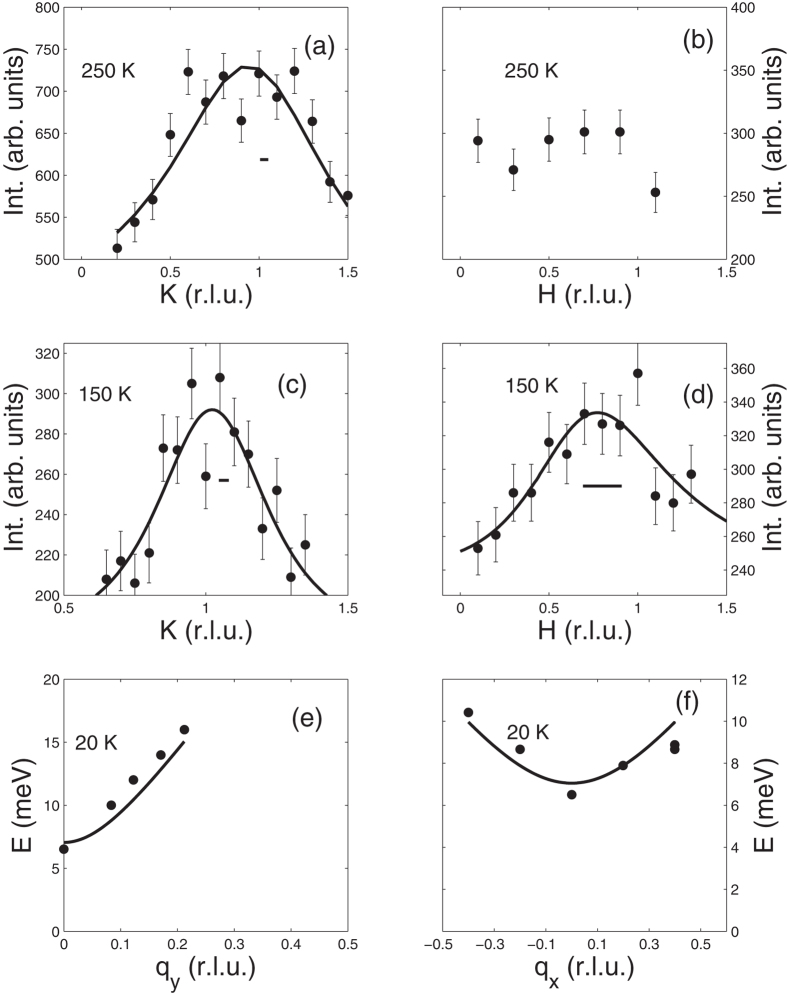
(**a**) Correlations along the chains measured at 250 K. (**b**) Correlations between chains measured at 250 K. (**c**) Correlations along the chains measured at 150 K. (**d**) Correlations between chains measured at 150 K. (**e**) Spin wave measurements along the chains measured at 20 K. (**f**) Spin wave measurements between the chains measured at 20 K. All fits described in the text. Horizontal lines indicate the instrument resolution.

Inelastic neutron scattering measurements of the small single crystal are shown in Fig. 6. In Fig. 6a,b, we showcase measurements made at 250 K of the intrachain and interchain coupling respectively. Due to spurious scattering at low energies, we measure the dynamic correlations at 3 meV and find a short correlation length of 1.8 ± 0.01 Å along the chains corresponding to the correlations between nearest neighbors only. No evidence of correlations between chains was found, as the scattering intensity is independent of *H* as shown in 6b. In Fig. 6c,d we look at the dynamic correlations at 150 K, measured at 8 meV. Here, the data is fit to the product of two 1D lorentzians convolved with the resolution function. We find a correlation length of 3.9 ± 1.5 Å along the chains and a correlation length of 2.8 ± 0.7 Å between the chains. This implies the formation of 2D correlations are developing above 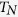, however, they are still very short range and confined to the nearest neighbors within a given plane. This is in sharp contrast with the extended correlations that develop below 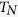 as demonstrated in Fig. 4.

To understand the excitations in the ordered phase, we consider the rectangular (also known as “quadratic”) Ising Hamiltonian^12^:


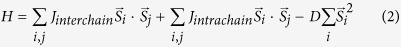


where 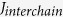 and 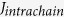 are interactions between and along chains respectively. *D* is a single-ion anistropy energy which is expected based on the appearance of a large spin-gap in both powder (Fig. 5) and single crystal (Fig. 6) samples. No interplane coupling between spins is considered in this model.

The spin wave dispersion was calculated from Eq. 2 using standard linear spin-wave theory in order to determine the exchange interactions from experiment. In Fig. 6e,f, we show the measured spin-wave dispersion along the reduced reciprocal lattice vectors 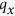 and 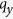 near the (0.526, 1, 0) magnetic reflection. The magnitude of the spin-gap observed at the magnetic zone center is consistent with that observed in the powder-averaged data in Fig. 5a. Fitting these dispersive excitations with the calculated spin-wave dispersion, shown as the solid line in the figures, we find 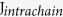 = 15.6 ± 1 meV, 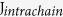 = 0.31 ± 0.1 meV, and D = 0.05 ± 0.01 meV. This confirms that the system is dominated by strong antiferromagnetic interactions along the chains, with weak coupling between them. The single-ion anisotropy is also consistent with our proposed spin structure with spins confined along the Jahn-Teller distorted *c*-axis. The large ratio 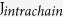/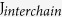 is consistent with the 1D-correlations observed above 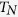 in both neutron scattering and magnetic susceptibility. Furthermore, the effective interaction determined from magnetic susceptibility is in remarkable agreement with 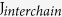 determined here.

## Discussion

To understand the physics of this system it is instructive to return to the lattice. At high temperatures (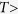 1323 K), ZnMn_2_O_4_ is a cubic spinel with magnetic sites forming a network of corner sharing tetrahedra such that all antiferromagnetic interactions between nearest neighbors are equal and frustrated. At lower temperatures the Jahn-Teller transition drives a cubic-to-tetragonal structural phase transition thereby increasing the distance of the interplane spins relative to those of the intraplane spins by over 7%. As the Mn-O octahedra are are connected by edges rather than corners, the dominant interactions will occur through direct exchange between the 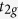 orbitals of the Mn ions^15^, and should exhibit strong dependence on the Mn-Mn separation distance. The structural phase transition therefore acts to increase the strength of the interactions in the a-b plane and weaken those out of the plane, effectively relaxing the frustration of the lattice from equilateral to isosceles tetrahedral geometry. In this lowered symmetry the interplane interactions remain frustrated, as can be seen in Fig. 3a. The ferro-orbital ordering^15^ resulting from the structural transition also contributes to the diminishing of interactions between the layers for the 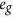 electrons.

Within each plane we find a large anisotropy in the strengths of the the interchain and intrachain couplings. This can be understood from a simple examination of the crystal structure, as the non-magnetic Zn-O tetrahedral sites lie between the Mn-O octahedral sites such that the interchain interactions are mediated mediated by the substantially weaker super- and super-super exchange interactions. As a result, the high temperature magnetic susceptibility and correlations are dominated by the much stronger direct exchange interactions along the chains within each layer giving rise to the 1D like behavior at high temperatures.

As we have shown, the ratio 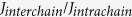 ∼ 0.02, and although small, is far larger than those found in more ideal 1D-chain systems^12^. This is sufficient to provide the necessary interaction between spins to generate 2D correlations in addition to the strong correlations along the chains. Long-range order in 2D, however, requires that there must be an anisotropy term which breaks the 3D rotational symmetry of the Heisenberg interaction^18^. In this system this is made possible by the uniaxial anisotropy along the *c*-axis produced by Jahn-Teller distortions. Together, this allows for 2D ordering of the lattice and suggests why the system remains two dimensional (with weak correlations along the c-axis) across such an extraordinarily large temperature range. The stabilization of two dimensional order has been observed in many other systems^22^,^23^,^24^,^25^, however, most either order with fully 3D structure at low enough temperature, or have chemical unit cells in which magnetic planes are vastly separated by non-magnetic layers. Thus, ZnMn_2_O_4_ presents a rare example of 2D ordering in a fully 3D crystal driven primarily by frustration.

In summary, we have studied the frustrated magnet ZnMn_2_O_4_ through bulk probes and neutron scattering. We have found that the interplay of orbital ordering and geometric frustration have given rise to a rare case of 2D ordering on the spinel lattice. The dynamical correlations unexpectedly retain low dimensionality over a wide temperature range, exhibiting an interesting series of dimensional crossovers from 1D to 2D within a 3D high symmetry lattice.

## Methods

Powder samples were prepared using the standard solid state synthesis method. Large single crystals were grown using the flux method. Powder neutron measurements were performed on the DCS time of flight spectrometer and BT1 diffractometers at NIST. Single crystal measurements were performed on the BT2, BT7, and BT9 triple axis spectrometers at NIST and the SCD time of flight Laue diffractometer at IPNS. The SCD measurements clearly show the absence of a-c twins. Further measurements were performed on the HB-3 spectrometer at HFIR.

## Additional Information

**How to cite this article**: Disseler, S. M. *et al*. One Dimensional(1D)-to-2D Crossover of Spin Correlations in the 3D Magnet ZnMn_2_O_4_. *Sci. Rep*. **5**, 17771; doi: 10.1038/srep17771 (2015).
